# Low Dark Current and Performance Enhanced Perovskite Photodetector by Graphene Oxide as an Interfacial Layer

**DOI:** 10.3390/nano12020190

**Published:** 2022-01-06

**Authors:** Ali Hassan, Muhammad Azam, Yeong Hwan Ahn, Muhammad Zubair, Yu Cao, Abbas Ahmad Khan

**Affiliations:** 1 International Science & Technology Cooperation Base for Laser Processing Robots, Wenzhou University, Wenzhou 325035, China; 15alirao@gmail.com (A.H.); yucao@wzu.edu.cn (Y.C.); 2 Department of Physics, Faculty of Sciences, University of Central Punjab, Lahore 54000, Pakistan; muhammad.azam01@ucp.edu.pk; 3 Department of Physics and Department of Energy Systems Research, Ajou University, Suwon 16499, Korea; ahny@ajou.ac.kr; 4 Department of Physics, Abbottabad University of Science and Technology, Abbottabad 22010, Pakistan; zubairphy@aust.edu.pk

**Keywords:** photodetector, perovskite, graphene oxide, low dark current, defect passivation

## Abstract

Organic-inorganic hybrid perovskite photodetectors are gaining much interest recently for their high performance in photodetection, due to excellent light absorption, low cost, and ease of fabrication. Lower defect density and large grain size are always favorable for efficient and stable devices. Herein, we applied the interface engineering technique for hybrid trilayer (TiO_2_/graphene oxide/perovskite) photodetector to attain better crystallinity and defect passivation. The graphene oxide (GO) sandwich layer has been introduced in the perovskite photodetector for improved crystallization, better charge extraction, low dark current, and enhanced carrier lifetime. Moreover, the trilayer photodetector exhibits improved device performance with a high on/off ratio of 1.3 × 10^4^, high responsivity of 3.38 AW^−^^1^, and low dark current of 1.55 × 10^−11^ A. The insertion of the GO layer also suppressed the perovskite degradation process and consequently improved the device stability. The current study focuses on the significance of interface engineering to boost device performance by improving interfacial defect passivation and better carrier transport.

## 1. Introduction

In recent years, photodetectors (PDs) have been attracting much interest due to their potential applications in the field of optical communication [1,2,3], large-area optoelectronic devices [1,2,3], environmental monitoring [4,5], day- and night-time surveillance [6,7,8], and biomedical sensing [9,10]. To date, most research consideration of photodetectors has been devoted to the vertical structures [11,12,13,14], whereas lateral photodetector architectures [15,16,17] have attracted increasing attention lately due to their ease of fabrication. A variety of promising materials, such as wide band gap semiconductor metal oxides [18,19], methylammonium lead halide perovskites [20], and transition metal dichalcogenides [9,21] have been used for lateral PDs. The metal oxide-based photodetector suffers from persistent photoconductivity due to their native defects which slow the photoresponse [22]. Conversely, the organic materials, including small molecules and polymers, exhibit great potential owing to their virtues of low-cost, simple, and flexible fabrication [17,23]. However, the photogenerated excitons in organic materials are difficult to be dissociated into free electrons and holes due to large binding energies (0.3–1.0 eV) [24]. In addition, the dissociated electrons still need to overcome the depletion barrier and require large diffusion length created by multilayer structure [25].

Numerous efforts have been made to overcome these obstacles, for instance, by incorporating small molecules [26], using an efficient electron transport layer [27], blending of acceptor and donor materials [28], and integrating conventional semiconductors such as metal oxides and functional 2D materials [14,19]. For instance, Chen et al. proposed the multilayer structure of organic/inorganic materials for attaining high responsivity in hybrid photodetectors [29]. The device exhibited an on/off ratio of 2600 with considerable responsivity of 22 mA/W; however, the device exhibited relatively high dark current (2.5 nA). Lee et al. [30] proposed a graphene–perovskite based bilayer photodetector which showed high responsivity of 180 A/W, but the device suffered from very poor on/off switching ratio with a slightly longer decay time. More recently, Xiaohui et al. reported the TiO_2_/perovskite bilayer photodetector; the proposed device exhibited considerably low dark current (1.05 × 10^−10^ A) with an on/off ratio of 4000 [31]. Until now, although the highly efficient carrier transport layer based on the low-dimensional material has proven to be effective in the fabrication of high speed perovskite photodetectors, the addition of graphene layer usually causes the increased dark current and relatively low switching ratio [30].

In the present study, we projected a planar photodetector with a hybrid structure of glass/TiO_2_/GO/MAPbI_3_/Al to minimize the dark current and to improve the on/off ratio. Here, the GO layer was used as an interfacial layer to reduce the leakage current while improving the other device parameters. The experimental results indicate that the insertion of GO as an interfacial layer not only enhances the overall device performance, but also helps to slow the perovskite degradation process, resulting in the enhanced device stability.

## 2. Materials and Methods

### 2.1. Materials Preparation

Graphene oxide was purchased from Nanjing XFNANO Materials Tech Co., Ltd., Nanjing, China. MAI and PbI_2_ were purchased from Xi’an Polymer Light Technology Corporation, Xi’an, China. DMF and DMSO were purchased from Alfa Aesar, Shanghai, China and TiO_2_ 10% soluble in ethanol (particle size ~30 nm) were purchased from InnoChem, Beijing, China. All prescribed materials were used as is (i.e., without any extra purification). Glass slides manufactured by SAIL BRAND, Hangzhou, China were used as substrate after three-step cleaning.

### 2.2. Solution Preparation

The anatase TiO_2_ was purchased from Innochem, Beijing, China. The TiO_2_ nanoparticles (NPs) were dispersed into Ethanol (1:16 volume ratio). To reduce the compactness of NPs of TiO_2_, a 50 µL TiO_2_ was dissolved into 800 µL ethanol and stirred for 1 h at room temperature.

By dissolving CH_3_NH_3_I and PbI_2_ with a molar ratio of 1:1 in *N*,*N*-dimethylformamide (DMF), and dimethyl sulfoxide (DMSO), a 1 M solution mixture was prepared. The prepared solution then continuously stirred at 60 °C for 12 h to obtain the homogeneity.

### 2.3. Device Fabrication

The glass substrates were cleaned sequentially with deionized water, acetone, and isopropanol with 20 min ultrasonication. Then, sonicated glass substrates were dried with Nitrogen air and given ozone treatment for 20 min for better crystallinity and defect-free growth area. The TiO_2_ layer was spin coated on these substrates at 4000 rpm for 30 s and annealed in ambient environment at 150 °C for 30 min. Afterward, GO was spin-coated on the TiO_2_ with 3000 rpm for the 40 s under inert environment and annealed at 130 °C for 10 min. Subsequently, MAPbI_3_ perovskite solution was deposited with the two-step method; in the first step the speed was adjusted at 1000 rpm for 10 s, and the second step was completed with the speed of 5000 rpm for 30 s. During the second step, the 50 µL toluene was dropped on the film at 20 s before the spin coating stopped and, later, aluminum (Al) metal electrodes of thickness 60 nm were deposited on GO film via thermal evaporation using a metal shadow mask. The shadow mask contains the channel width of 2000 µm and a channel length of 30 µm.

### 2.4. Characterization

A Keithley 4200 (Keithley Instruments, LLC, Solon, OH, USA) was used for all electrical device characterization at room temperature and the ambient environment. An XZ-150WA Halogen lamp of cold light illuminator (Nanjing, China) was used for white light illumination. The mono silicon detector was utilized for calibration of the spectral range and light intensity of white light before usage. The Olympus BX51 optical microscope (Olympus Corporation, Tokyo, Japan) was used to collect the optical image of the device. The surface images and morphological study of the films were carried out using SEM (Hitachi S-4800, Tokyo, Japan). Raman measurements were taken using a T64000 confocal Raman spectrometer Horiba-JY iHR550 (Horiba, Kyoto, Japan) with a 532 nm excitation laser in back scattering configuration. Visible spectra, steady-state PL, and time-resolved PL of films were measured using JASCO-570, JASCO FP 6600 (JASCO, Tokyo, Japan), and FLS980 fluorescence spectrometer system (Edinburgh Instruments Ltd. Livingston, UK), respectively. The ultraviolet photoelectron spectroscopy (UPS) was carried out using a KRATOS AXIS ULTRA (KRATOS, Kyoto, Japan) delay-line detector photoelectron spectroscopy with an unfiltered He-I (21.22 eV) gas-discharge lamp. The phase identification was analyzed using a Rigaku D/MAX-2004 (Rigaku Corporation, Tokyo, Japan) XRD with Cu K radiation (λ = 1.54178 Å) which is operates at 40 kV and 60 mA.

## 3. Results and Discussions

**Figure 1:**
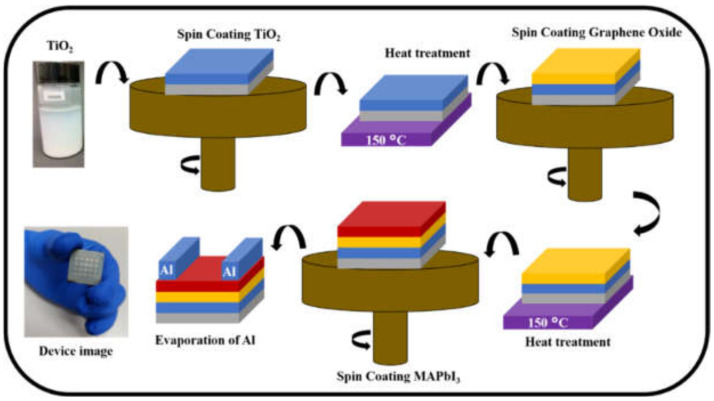
Schematic illustration of the device fabrication process and optical image of the fabricated device.

Figure 1 shows the schematic illustration of the whole fabrication process of the trilayer perovskite photodetector. An optical image of the fabricated device is also shown in Figure 1. The trilayer photodetector was fabricated on glass substrate using two-step spin-coating technique with toluene as an anti-solvent. The MAPbI_3_ perovskite was dissolved in N, N-dimethylformamide (DMF), and DMSO, while graphene oxide (GO) was dispersed in water as previously reported [32,33]. Firstly, TiO_2_, as an electron transport layer (ETL), was deposited on the substrate and annealed, followed by the GO layer deposition using a spin-coating method. Afterward, the perovskite layer was deposited on the GO layer. Subsequently, the aluminum metal (Al) electrodes were evaporated on the perovskite layer using an interdigital mask through thermal evaporation. The channel width and length were 2000 µm and 30 µm, respectively.

**Figure 2:**
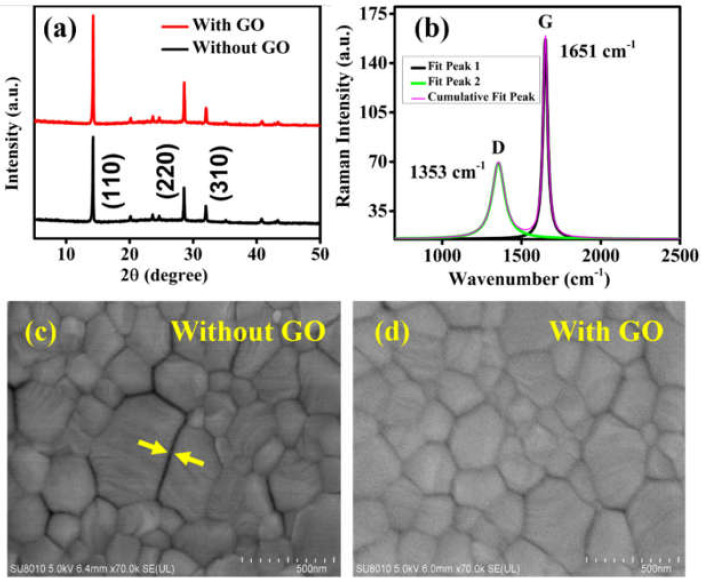
(**a**) XRD spectra of perovskite films with and without graphene oxide, and (**b**) Raman spectrum of the GO. The Raman spectrum fitted with Lorentz function, having typical D and G bands that are associated with graphite material. FE-SEM micrograph of the perovskite films (**c**) without GO interlayer having wide gaps, and (**d**) with GO interlayer having narrow and comparatively fewer gaps.

X-ray diffraction (XRD) study has been carried out for observation of the crystallinity of single layer (without GO) and trilayer (with GO) perovskite films, as shown in Figure 2a. The perovskite film without GO exhibits the characteristic XRD peaks at 14.24, 28.60, and 31.96 degrees, which represent the (110), (220), and (310) lattice planes, respectively [34,35]. More interestingly, no PbI_2_ residual peak has been found in both (with and without GO) perovskite films. The intensity of (110) peak in film with GO is higher as compared to the film without GO. Moreover, the crystallite size has been calculated using the well-known Scherrer formula; interestingly, the perovskite layer with GO modifier exhibited slightly higher crystallite size (65 nm) as compared with the perovskite layer without GO (57.89 nm). This states that the crystallinity of perovskite film has been improved noticeably by an interfacial graphene oxide layer which is also in good accordance with the field emission scanning electron microscopy (FE-SEM) analysis as will be discussed in Figure 2c,d. Figure 2b shows the Raman spectra of graphene oxide film, as the Raman spectroscopy is one of the significant tools to identify and characterize graphene-related materials. The typical graphite D and G bands (D ~ 1353 cm^−1^, G ~ 1651 cm^−1^) in graphene oxide film have been observed. The strong active G band represents the in-phase graphite lattice vibrations, whereas the D bands indicate the disorder and defects in the lattice [36]. The graphene oxide was deposited on the glass substrate to perform the Raman analysis. The slight Raman shift in the G band towards higher wavenumber is likely due to the relatively low concentration of solution (0.5 mg/mL) used to spin-coat GO layer, which is beneficial to reduce the dark current [37].

The corresponding interfacial passivation not only improves the crystallinity of perovskite film, but also enhance the quality of the film. Figure 2c,d show the top-scan views of FE-SEM images of perovskite without and with graphene oxide interfacial layer, respectively. By introducing a GO interfacial layer, the wide gaps or cracks in the grain boundaries were reduced significantly. The FE-SEM image of the graphene oxide layer has been shown in Figure S1 of Supplementary Materials. The planarization effect introduced by the GO film could provide a better interface for perovskite layer, leading to the optimal growth of perovskite film with large grain size, and hence, with the improved crystallinity. The pinholes, grain boundaries, and cracks on the perovskite film surface could work as a non-radiative recombination centers and electron traps [38]. Therefore, the corresponding interfacial defect treatment by GO layer not only improves the uniformity of the perovskite film but also reduces the structural abnormalities that have been caused by cracks and pinholes. Obviously, this will improve the device performance significantly by minimizing the interband charge recombination at interfaces and by providing a high carrier transport rate [38,39].

**Figure 3:**
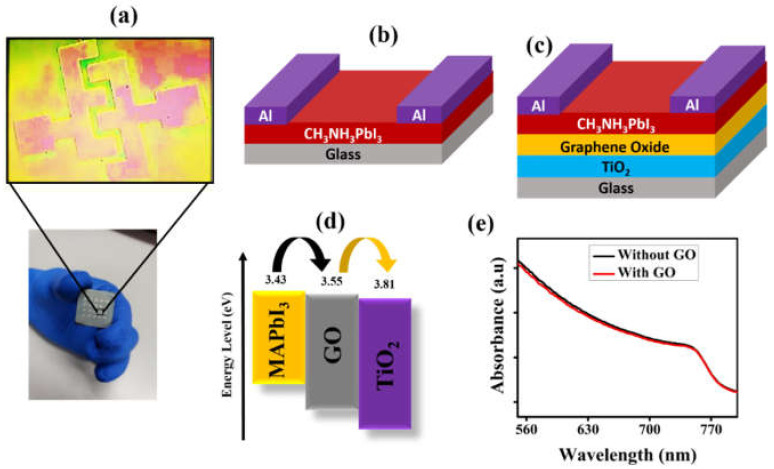
(**a**) Optical image of the device showing the metal contacts. Schematic cross section of (**b**) the single layer (without GO) device, and (**c**) the trilayer (with GO) device. (**d**) Schematic energy level diagram of the trilayer structure. (**e**) Absorbance spectra of perovskite (with and without GO) films.

Figure 3a shows a photographic image of the fabricated photodetector. Figure 3b,c represent the schematic device structure of the single layer and trilayer photodetectors, respectively. Figure S2 in the Supplementary Materials shows the bilayer schematic structure of the photodetectors. We would like to note that the graphene oxide improves the morphology of the perovskite film as mentioned above but, more importantly, it works as an interfacial layer to extract more charge carriers from the active layer to enhance device performance significantly [29,30]. The energy band diagram of ETL, perovskite, and graphene oxide has been shown in Figure 3d. The band energy levels were calculated from the band edge values from the UPS measurements combined with the optical bandgap (1.58 eV) (see the Supplementary Materials Figures S3 and S4). The conduction band minima (CBM) for the ETL layer was found at 3.81 eV, whereas the CBM for graphene oxide and perovskite film was 3.55 eV and 3.43 eV, respectively. It is obvious that the energy barriers for electron transport from the perovskite to the TiO_2_ layer is more efficient by introducing GO layer, as it reduces the barrier height significantly. Consequently, the introduction of graphene oxide (3.55 eV) helps the fast transfer of electrons across the TiO_2_ layer and further reduces the energy loss of charge carriers across the interface of trilayer photodetector [40,41]. In other words, the compatible band alignment exhibits favorable conditions for the separation of photo-generated charge carrier with much fewer interfacial charge recombination. In addition, the respective UV–vis absorption spectra shown in Figure 3e indicate that the absorption of the trilayer (with GO) is almost the same as that of the single layer (without GO) perovskite film. This indicates that the perovskite layer only contributes the absorption, whereas the TiO_2_ and GO layers do not show any absorption.

**Figure 4:**
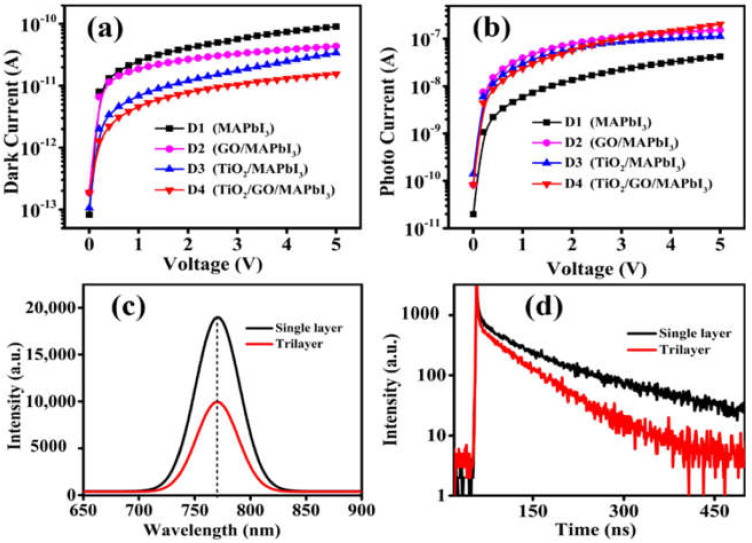
*I*–*V* curves of D1 (MAPbI_3_), D2 (GO/MAPbI_3_), D3 (TiO_2_/MAPbI_3_), and D4 (TiO_2_/GO/MAPbI_3_) trilayer devices (**a**) under dark and (**b**) under white light illumination of 0.1 mWcm^−2^. (**c**) Photoluminescence (PL) spectra, (**d**) time-resolved PL of single layer (without GO) and trilayer (with GO) film.

Figure 4a,b represent the current-voltage (*I*–*V*) curves of the different types of devices under dark and light illumination of 0.1 mWcm^−2^, respectively. In addition to the single layer (D1) and trilyer (D4) devices, we added two more devices for comparison, i.e., the bilayer perovskite devices with glass/GO/Perovskite (D2) and another bilayer device with glass/TiO_2_/Perovskite (D3). The D1 device shows the highest dark current of 9.03 × 10^−11^ A, at a bias voltage (*V*) of 5 V, while the D4 device with graphene oxide exhibits the lowest dark current of 1.55 × 10^−11^ A. The significant reduction in the dark current of the D4 device can be attributed to the presence of depletion region (caused by the addition of GO layer in our case) It is consistent with the fact that the dark current measured in the two bilayer perovskite devices reaches 4.35 × 10^−11^ A and 3.39 × 10^−11^ A, for D2 (GO/Perovskite) and D3 (TiO_2_/Perovskite), respectively, which are higher than that of D4 photodetector device. In other words, the low dark current in the D4 photodetector is caused by the extra depletion regions at the two interfaces introduced by GO layer, which leads to the narrowing of the conducting region [35].

As shown in Figure 4b, the photocurrent measured for D1 (without GO) device was 4.19 × 10^−8^ A, while the photocurrent of D4 (with GO) device was 2.02 × 10^−7^ A at *V* = 5 V. The high photocurrent for the D4 photodetector device is due to the heterojunctions created between the perovskite/graphene oxide and graphene oxide/TiO_2_, which helps the efficient charge transfer, as discussed above [42,43]. As the heterojunction spatially separates the holes and electrons, the hole remains in the perovskite layer, while the TiO_2_ ETL layer catches the electrons efficiently. Furthermore, the graphene oxide layer boosts the charge extraction process when the device is exposed to light illumination by providing some extra charge carriers from the active layer to the TiO_2_ layer. This will result in the further decrease in the carrier recombination at the interfaces, improving the device performance. Obviously, the improved morphology, such as the reduction in the pinholes and cracks (as discussed in Figure 2) would have contributed to the enhanced photocurrent in the trilayer structure [34,44]. The ratio of photocurrent to dark current (*I*_light_/*I*_dark_) has been calculated for all four devices at the light illumination intensity of 0.1 mWcm^−2^, and a bias voltage of 5 V. The calculated values of *I*_light_/*I*_dark_ for the devices have been shown in Table S1 of the Supplementary Materials. The trilayer device shows the highest on/off ratio (1.3 × 10^4^) which is increased 28-fold from that of the single layer device (464).

To investigate the optoelectronic properties and carrier recombination process of perovskite photodetectors tailored by the GO layer incorporation, the steady-state photoluminescence (PL) spectra and time-resolved photoluminescence (TRPL) of the single layer and trilayer films were measured. Figure 4c shows the PL spectrum of trilayer decreased by two-fold as compared to that of the single layer perovskite film under the same excitation intensity. The reduced PL intensity for trilayer film indicates the suppression of e-h (electron-hole) recombination and potentially the enhanced charge extraction efficiency of perovskite layer on GO layer [45]. This is as the presence of graphene oxide layer enhances the charge extraction process and reduces the undesired recombination, owing to the modified energy band alignment as discussed above in Figure 3d [38]. The TRPL results shown in Figure 4d are also consistent with the PL results. It is clear that a significant decrease in the PL lifetime of the trilayer indicates that the addition of GO significantly suppresses the carrier recombination and enhances the electron extraction from the perovskite layer to the interface through the GO layer [46,47]. These findings suggest that the photogenerated charge carriers in the perovskite layer were transferred more quickly to the GO layer which has a higher work function. The above results indicate that the rapid carrier collection prevents the charge accumulation at the perovskite and GO interface [48].

**Figure 5:**
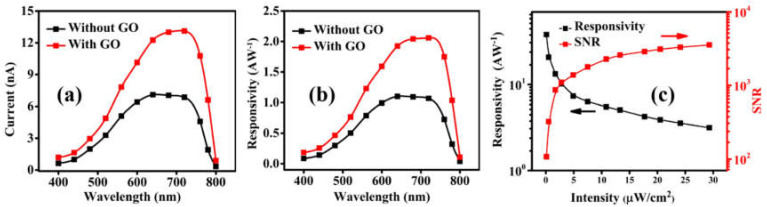
(**a**) Photo current spectra, (**b**) responsivity spectra of the single layer and the trilayer photodetector measured with a light illumination of 10.6 µW/cm^2^ and a bias voltage of 5 V. (**c**) The signal to noise ratio and responsivity of the trilayer photodetector as functions of illumination power of incident white light.

Figure 5a plots the spectral photocurrent of a single perovskite device and the trilayer device at *V* = 5 V. The trilayer device demonstrates higher photocurrent as compared to the single layer perovskite photodetector device over the entire wavelength range. To check the sensitivity of a photodetector, we calculated the responsivity (*R*) according to the following Equation (1):

(1) $$R=\frac{I_{light}-I_{dark}}{P_{in}}$$

where $I_{light}$ represents the current under light illumination, $I_{dark}$ represents the current without light illumination, and $P_{in}$ stands for the incident light power on an effective area [35]. According to the above relation, Figure S5 in Supplementary Materials shows that the *R* of a device will increase by increasing the bias voltage. Therefore, the calculated *R* for the single layer device and the trilayer device under white light illumination are 0.69 AW^−1^ and 3.38 AW^−1^, respectively.

The spectral current and responsivity of the single layer device and the trilayer device measured at different illuminating wavelength with an intensity of 10.6 µWcm^−2^ are shown in Figure 5a,b. The trilayer device with GO layer shows the higher responsivity as compared to the single layer perovskite device in the wavelength range of 400 to 800 nm. We also calculated signal-to-noise ratio (*SNR*) which is one of the important parameters to characterize the photodetector device with the following relation:

(2) $$SNR=\frac{I_{light}-I_{dark}}{I_{dark}}$$

The calculated *SNR* values for single layer and trilayer devices are 462 and 1.3 × 10^4^, respectively. Figure 5c shows the *SNR* and responsivity of the trilayer device as functions of the illuminating light intensity. The *SNR* increases in the high intensity condition owing to the large photocurrent generation, whereas the responsivity decreases with the intensity [49]. However, with the increase in the light intensity, the portion of photogenerated carriers that can be extracted reduces.

Linear dynamic range (*LDR*) is another crucial parameter to calculate the range of incident illumination power, which is defined by

(3) $$LDR=20log\frac{J_{light}}{J_{dark}}$$

where $J_{light}$ and $J_{dark}$ are the photocurrent and the dark current densities, respectively [50]. The single layer device shows the *LDR* value of 53.33 dB whereas the trilayer device shows the highest value of 82.30 dB among all four devices. The improved responsivity with higher *SNR* and *LDR* value of trilayer device points towards the clear advantage of graphene oxide for better performance of hybrid perovskite photodetectors.

**Figure 6:**
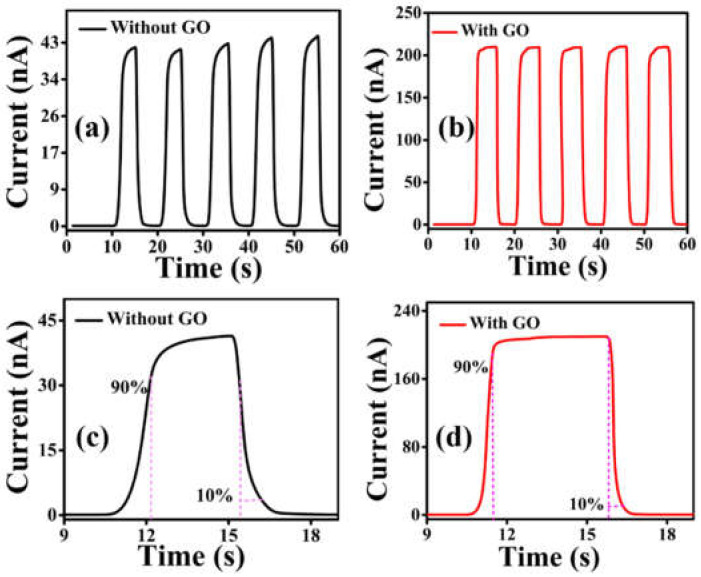
Time-dependent photocurrent response of (**a**) single layer (without GO) and (**b**) trilayer (with GO) photodetector measured at a bias voltage of 5 V and illumination power of 0.1 mWcm^−2^. One cycle photo response of (**c**) the single layer device and (**d**) the trilayer device for extraction of the rise and decay time.

Figure 6a,b show the on–off switching characteristics of the single layer and the trilayer devices, respectively. Both devices show good on–off switching repetition under light illumination of 0.1 mWcm^−2^. Figure S6 of Supplementary Materials shows the on–off switching behavior of the bilayer devices (D2 and D3). It has been noted that the switching property of the trilayer device is far better among all devices. The calculated rise and decay times for the single layer device were 1.15 s and 0.78 s, respectively, whereas, for the trilayer device, the rise and decay times were 0.45 s and 0.33 s, respectively. The improved time response in the trilayer device can be attributed to the reduced trap states. The single layer device contains large number of traps and wide gaps as shown in Figure 2c, which tends to create a barrier for the charge transport. It takes more time to fill these trap states; as a result, the rise time for the single layer device increases.

Conversely, under light illumination, it requires time for photogenerated carriers to fill these traps. Therefore, the photocurrent increases slowly to a steady value. By switching off the light, the decay time is prolonged, as it takes time for the carriers to be released from these traps [51,52]. In the trilayer photodetector, due to the presence of fewer trap states, the current attains its maximal value more quickly; on the other hand, while switching off the illumination, the decay occurs quickly. Consequently, the rise and decay time of the trilayer is much smaller than that of the single layer device which is beneficial for the high-speed devices [24].

**Figure 7:**
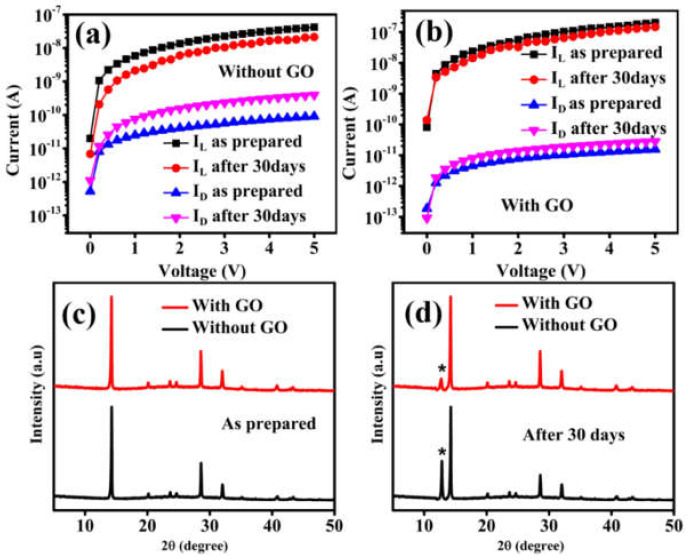
*I*–*V* curves of (**a**) the single layer (without GO) device and (**b**) the trilayer (with GO) device, measured as-prepared and after 30 days in an ambient environment. The samples were illuminated with the white light of illumination power 0.1 mW/cm^2^ and a bias voltage up to 5 V. XRD spectra of (**c**) as prepared, (**d**) after 30 days in an ambient environment, of the single layer and the trilayer films, here (*) represents the residual peak of PbI_2_.

To investigate the stability of the photodetector device, the samples were placed in an ambient environment for 30 days with a relative humidity of 40–50%. Figure 7a,b shows the *I*–*V* curves of the single layer and trilayer devices, respectively, measured as-prepared and after 30 days. The degradation of the single layer device, i.e., the increase in dark current and the decrease in photocurrent, is more obvious than that of the trilayer device. The detailed *I*–*V* stability parameters for single layer and trilayer device has been shown in Table S2. Figure 7c,d show the XRD spectra of films measured as prepared and after 30 days, respectively, for both the single layer (black line) and trilayer (red line). The trilayer film has a lower PbI_2_ peak as compared to the single layer device after 30 days, which indicates the trilayer device still has better crystallinity even after 30 days in an ambient environment. These results indicate that the interfacial GO layer played a vital role in slowing down the perovskite film degradation (due to the improved morphology) that occurs in the perovskite film after exposure to the ambient environment [53,54]. A detailed comparison of our trilayer device with previously reported photodetectors has been presented in Table S3. In terms of overall device performance, the proposed trilayer device shows a high on/off ratio, low dark current, and high responsivity.

## 4. Conclusions

In summary, a hybrid glass/TiO_2_/GO/MAPbI_3_/Al photodetector with a graphene oxide interfacial layer has been fabricated and investigated in detail. The insertion of GO interfacial layer enhances the performance of the hybrid perovskite photodetector, such as to achieve low dark current (~1.55 × 10^−11^ A), high on/off ratio (1.3 × 10^4^), and high responsivity (3.38 AW^−1^). The GO layer also suppresses the degradation of the perovskite layer. The trilayer photodetector device stability in an ambient environment shows high significance in future optoelectronic devices.

## Data Availability

The data is contained within the article or Supplementary Materials.

## Supplementary Materials

The following supporting information can be downloaded at: www.mdpi.com/article/10.3390/nano12020190/s1, Figure S1: SEM image of graphene oxide layer on glass substrate; Figure S2: Schematic diagram of bilayer photodetector device containing (a) TiO2/MAPbI3 (b) GO/MAPbI3; Figure S3: Ultraviolet Photoelectron Spectroscopy (UPS) spectra for (a) TiO2, (b) Perovskite (MAPBI3) and (c) Graphene Oxide layer; Figure S4: Tauc’s plot of single layer (without GO) and trilayer (with GO) films derived from the absorbance data; Figure S5: Responsivity of single layer (without GO) and trilayer (with GO) devices with increasing voltage. The responsivity increases as the bias voltage increases. The trilayer device exhibit higher responsivity; Figure S6: (a,b) On/Off switching results of bilayer devices (GO/MAPbI3 and TiO2/MAPbI3). Single cycle with rise and decay time of (c) GO/MAPbI3 (d) TiO2/MAPbI3; Table S1: Device results of all four devices for detailed comparison; Table S2: Stability parameters of Single and Trilayer device after 30 days in ambient environment; Table S3; Comparison of Single, Bilayer, and Trilayer photodetector device parameters previously reported with current study. Refs. [29,30,35,51,55,56,57,58,59,60,61,62,63,64,65] are cited in the Supplementary Materials.
