# Supplementary material for: Low Dark Current and Performance Enhanced Perovskite Photodetector by Graphene Oxide as an Interfacial Layer
Source: Nanomaterials (Basel). 2022 Jan 6;12(2):190. doi: 10.3390/nano12020190 (PMC8778836; doi:10.3390/nano12020190)
Supplement: Supplementary file 1 [file nanomaterials-12-00190-s001.zip › nanomaterials-1524442-supplementary.pdf]

nanomaterials

Supplementary Materials

Low Dark Current and Performance Enhanced Perovskite Photodetector by Graphene Oxide as an Interfacial Layer

Ali Hassan [1], Muhammad Azam [2], Yeong Hwan Ahn [3], Muhammad Zubair [4], Yu Cao [1], and Abbas Ahmad Khan [3,*]

Citation: Hassan, A.; Azam, M.; Ahn, Y.H.; Zubair, M.; Cao, Y.; Khan, A.A. Low Dark Current and Performance Enhanced Perovskite Photodetector by Graphene Oxide as an Interfacial Layer. *Nanomaterials* **2022**, *12*, 190. https://doi.org/10.3390/nano12020190

Academic Editor: Alain Pignolet

Received: 9 December 2021
Accepted: 4 January 2022
Published: 6 January 2022

Publisher's Note: MDPI stays neutral with regard to jurisdictional claims in published maps and institutional affiliations.

Copyright: © 2022 by the authors. Licensee MDPI, Basel, Switzerland. This article is an open access article distributed under the terms and conditions of the Creative Commons Attribution (CC BY) license (https://creativecommons.org/licenses/by/4.0/).

[1] International Science & Technology Cooperation Base for Laser Processing Robots, Wenzhou University, Wenzhou 325035, China; 15alirao@gmail.com (A.H.); yucao@wzu.edu.cn (Y.C.)
[2] Department of Physics, Faculty of Sciences, University of Central Punjab, Lahore 54000, Pakistan; muhammad.azam01@ucp.edu.pk
[3] Department of Physics and Department of Energy Systems Research, Ajou University, Suwon 16499, Korea; ahny@ajou.ac.kr
[4] Department of Physics, Abbottabad University of Science and Technology, Abbottabad 22010, Pakistan; zubairphy@aust.edu.pk
* Correspondence: abbaskhan@ajou.ac.kr

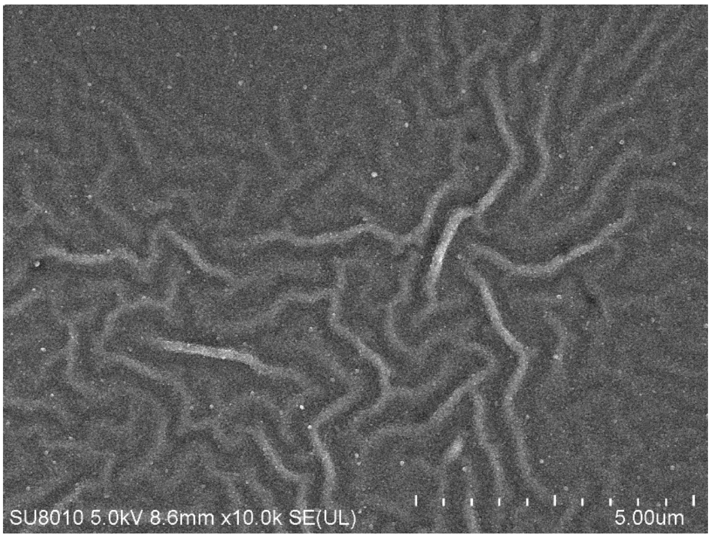

Figure S1. SEM image of graphene oxide layer on glass substrate.

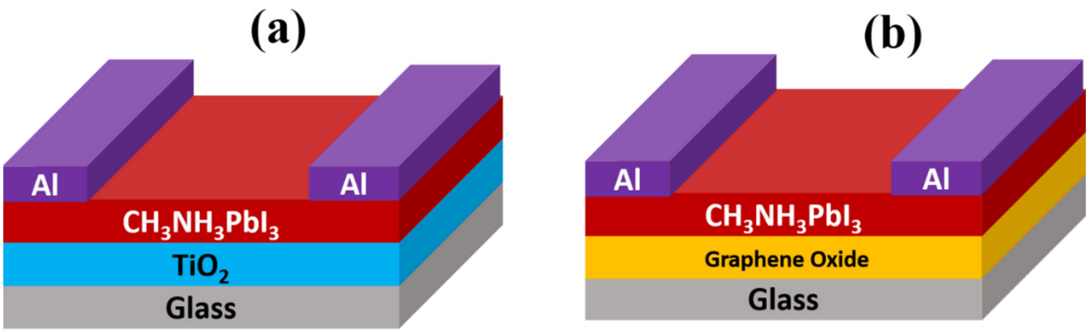

Figure S2. Schematic diagram of bilayer photodetector device containing (**a**) $TiO_2/MAPbI_3$ (**b**) $GO/MAPbI_3$.

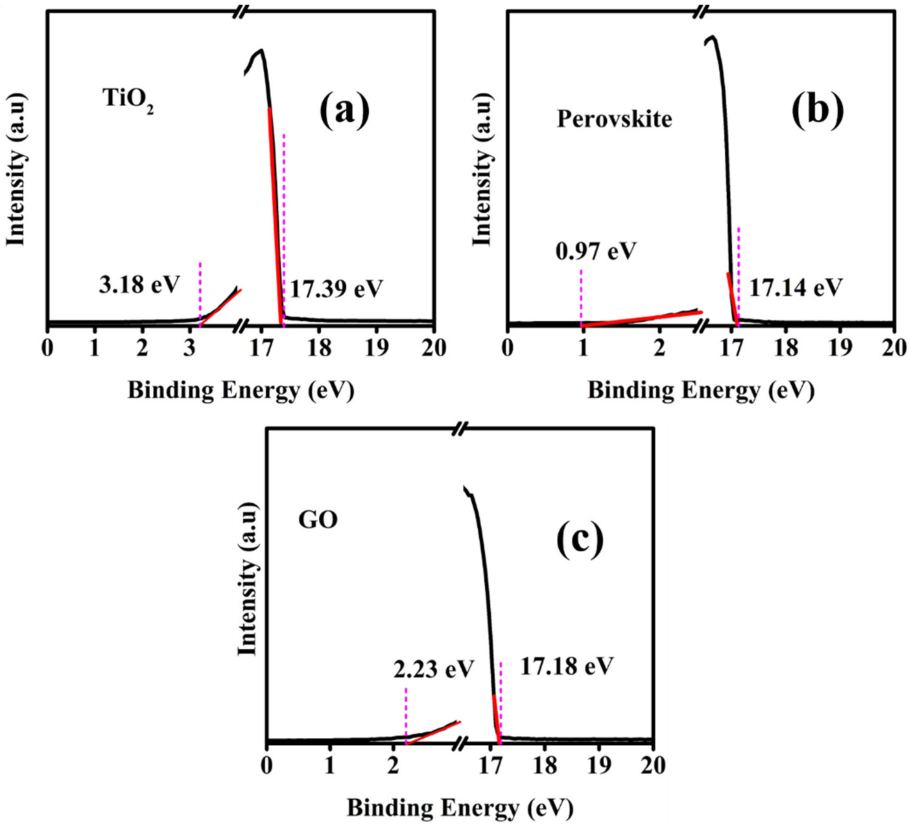

Figure S3. Ultraviolet Photoelectron Spectroscopy (UPS) spectra for (**a**) TiO_2, (**b**) Perovskite ($MAPBI_3$) and (**c**) Graphene Oxide layer.

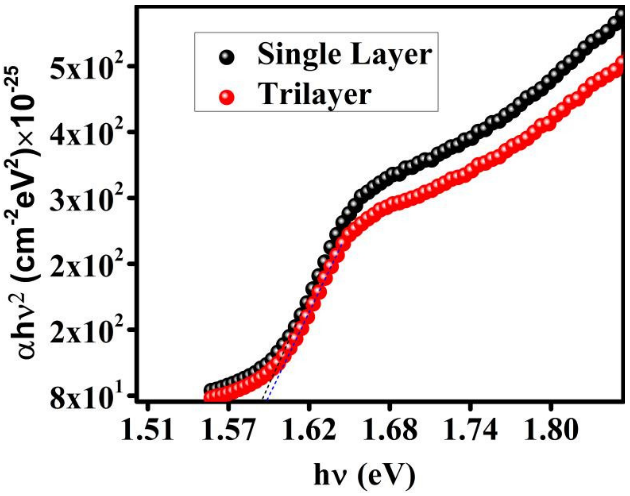

Figure S4. Tauc's plot of single layer (without GO) and trilayer (with GO) films derived from the absorbance data.

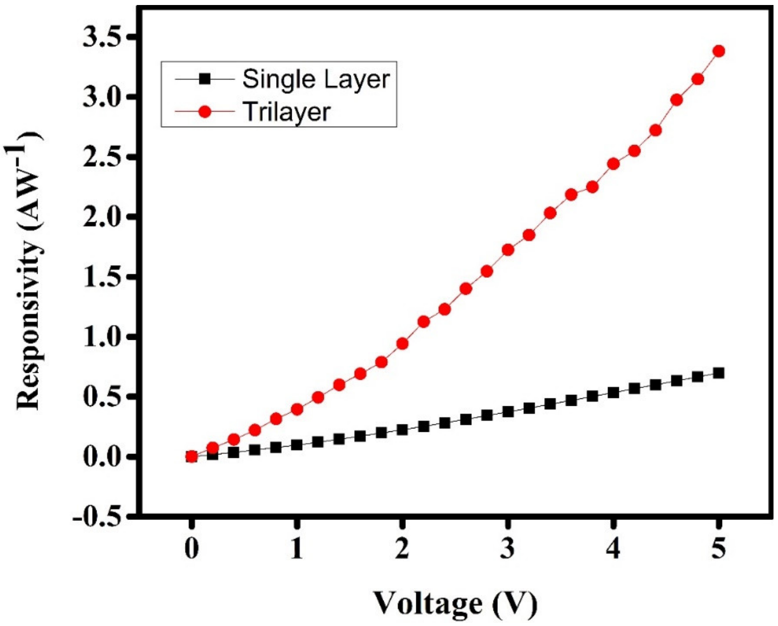

Figure S5. Responsivity of single layer (without GO) and trilayer (with GO) devices with increasing voltage. The responsivity increases as the bias voltage increases. The trilayer device exhibit higher responsivity.

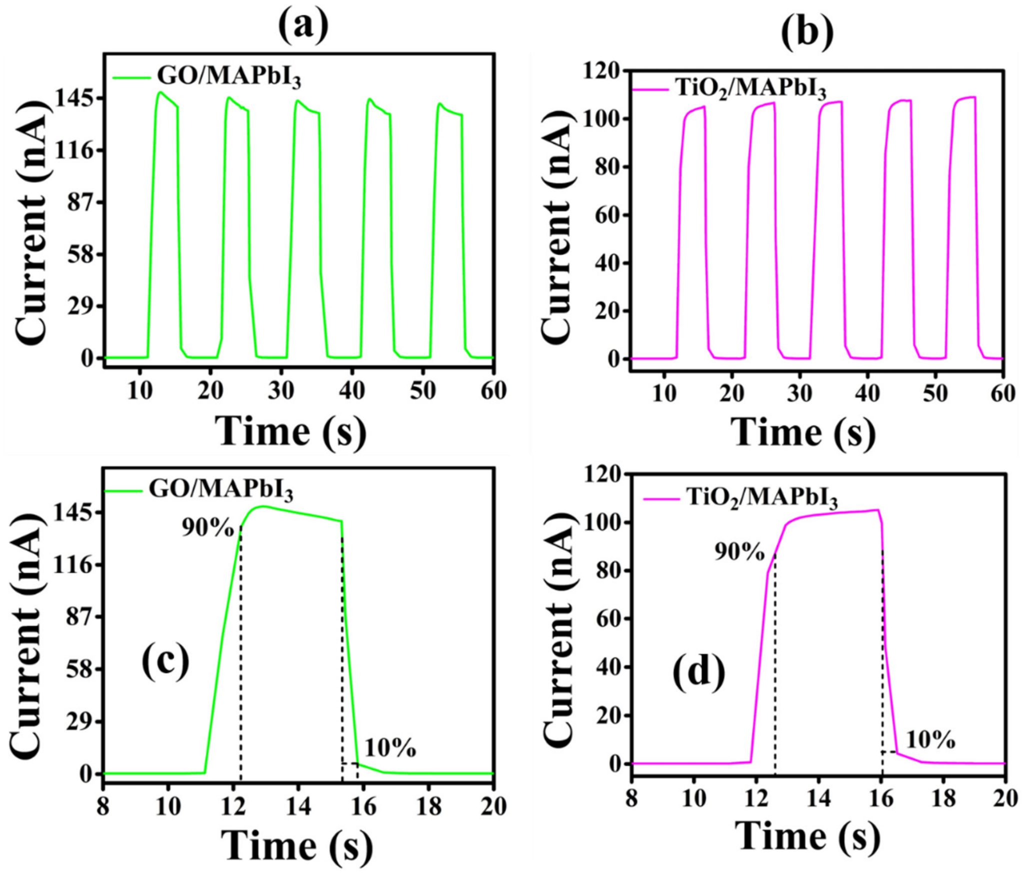

Figure S6. (**a**,**b**) On/Off switching results of bilayer devices ($GO/MAPbI_3$ and $TiO_2/MAPbI_3$). Single cycle with rise and decay time of (**c**) $GO/MAPbI_3$ (**d**) $TiO_2/MAPbI_3$.

Table S1. Device results of all four devices for detailed comparison.

Device	Dark Current (A)	Light Current (A)	Responsivity (A/W)	On/off ratio	Rise/decay (s)
D1 ($MAPbI_3$)	9.03×10^{-11}	4.19×10^{-8}	0.69	464	1.15/0.78
D2 ($GO/MAPbI_3$)	4.35×10^{-11}	1.52×10^{-7}	2.54	3494	0.98/0.43
D3 ($TiO_2/MAPbI_3$)	3.39×10^{-11}	1.11×10^{-7}	1.86	3274	0.95/0.42
D4 ($TiO_2/GO/MAPbI_3$)	1.55×10^{-11}	2.02×10^{-7}	3.38	13,031	0.45/0.33

Table S2. Stability parameters of Single and Trilayer device after 30 days in ambient environment.

Device	Dark Current (A)	Light Current (A)	Responsivity (A/W)	On/off ratio
D1 ($MAPbI_3$)	3.99×10^{-10}	4.18×10^{-8}	0.65	105
D4 ($TiO_2/GO/MAPbI_3$)	2.84×10^{-11}	1.46×10^{-7}	2.43	5140

Table S3. Comparison of Single, Bilayer, and Trilayer photodetector device parameters previously reported with current study.

Structure	Wavelength (nm)/Illumination Power	Dark Current (A)	On/Off ratio	R (A/W)	Rise/Decay Time	Active Area (μm^2)	Ref.
$MAPbI_3$	$550/2.7 \times 10^{-3}$ mW	1.67×10^{-9}	4.89	0.027	1.2/0.2 s	10,000 × 15,000	[1]
$MAPbBr_3$	532/100 mW	1.0×10^{-7}	-	0.1	70/150 μs	-	[2]
$PCBM/MAPbI_3$	365/2 mW	10-7	3000	0.18	123/180 ms	-	[3]
$PDPP3T/MAPbI_3$	365/0.5<br>650/0.5<br>937/0.5	84.5×10^{-9}	6<br>15<br>33	0.0107<br>0.0255<br>0.0055	50/100 ms<br>40/140 ms<br>30/150 ms	330,000	[4]

Single Crystal $MAPbI_3$	780/0.32<br>780/0.05<br>405/0.05	4×10^{-8}	<4000<br><1000<br><500	1.6<br>2.531<br>0.181	-	85,000	[5]
Nanocrystals $CsPbI_3$	405/1.38	$\sim 1 \times 10^{-9}$	100,000	0.31	24/29 ms	3 × 7800	[6]
$MAPbI_3$/graphene	520/2	$\sim 0.5 \times 10^{-3}$	<1.5	180	0.087/0.54 s	50 × 1000	[7]
$CH_3NH_3I_3$/graphene/Au NPs	532/0.014	$\sim 8.8 \times 10^{-4}$	<2	2.1 × 103	1.5/10 s	40 × 20,000	[8]
Graphene/Psk/Graphene	452/2.1	2.5×10^{-9}	2.6×10^{3}	0.022	-	-	[9]
$MAPbIBr_2$/Graphene	405/1.052 nW	5.5×10^{-3}	~1.2	6×10^{5}	0.12/0.75 s	-	[10]
NWs $MAPbI_3$/graphene	$633/6.5 \times 10^{-5}$	$\sim 2.7 \times 10^{-7}$	~1.2	2.6×10^{6}	55/75 s	10 × 10	[11]
$MAPbI_3/MoS_2$	520/6<br>655/6<br>785/6	$\sim 1.5 \times 10^{-5}$	~10,000<br>~6000<br>~1000	~2120<br>~824<br>~102	6.17/4.5 s	5 × 5	[12]
$MAPbI_3/WS_2$	505/0.5	$\sim 10^{-10}$	$\sim 3 \times 10^{5}$	2.4	2.7/7.5 ms	10 × 2000	[13]
$MAPbBr_3$/EA/TiO_2	White/0.5	1.51×10^{-11}	2700	0.13	0.49/1.17 s	30 × 2000	[14]
GQDs/$MAPbI_3$	405/1.6	4.5×10^{-5}	-	12	140/160 ms	-	[15]
**This Work**	White/0.1	$\sim 1.55 \times 10^{-11}$	1.3×10^{4}	3.38	0.45/0.33 s	30 × 2000	-

Note: Psk here represents the perovskite.

References

1. Lu, H.; Tian, W.; Cao, F.; Ma, Y.; Gu, B.; Li, L. A Self-Powered and Stable All-Perovskite Photodetector-Solar Cell Nanosystem. *Adv. Funct. Mater.* **2016**, *26*, 1296–1302, https://doi.org/10.1002/adfm.201504477.
2. Shaikh, P.A.; Shi, D.; Retamal, J.R.D.; Sheikh, A.D.; Haque, A.; Kang, C.-F.; He, J.-H.; Bakr, O.M.; Wu, T. Schottky junctions on perovskite single crystals: light-modulated dielectric constant and self-biased photodetection. *J. Mater. Chem. C* **2016**, *4*, 8304–8312, https://doi.org/10.1039/c6tc02828d.
3. Wang, Y.; Zhang, T.; Zhang, P.; Liu, D.; Ji, L.; Chen, H.; Chen, Z.D.; Wu, J.; Li, S. Solution processed PCBM-$CH_3NH_3PbI_3$ heterojunction photodetectors with enhanced performance and stability. *Org. Electron.* **2018**, *57*, 263–268, https://doi.org/10.1016/j.orgel.2018.02.043.
4. Chen, S.; Teng, C.; Zhang, M.; Li, Y.; Xie, D.; Shi, G. A Flexible UV–Vis–NIR Photodetector based on a Perovskite/Conjugated-Polymer Composite *Adv. Mater.* **2016**, *28*, 5969–5974, https://doi.org/10.1002/adma.201600468.
5. Ding, J.; Du, S.; Zhao, Y.; Zhang, X.; Zuo, Z.; Cui, H.; Zhan, X.; Gu, Y.; Sun, H. High-quality inorganic–organic perovskite $CH_3NH_3PbI_3$ single crystals for photo-detector applications. *J. Mater. Sci.* **2016**, *52*, 276–284, https://doi.org/10.1007/s10853-016-0329-2.
6. Ramasamy, P.; Lim, D.-H.; Kim, B.; Lee, S.-H.; Lee, M.-S.; Lee, J.-S. All-inorganic cesium lead halide perovskite nanocrystals for photodetector applications. *Chem. Commun.* **2015**, *52*, 2067–2070, https://doi.org/10.1039/c5cc08643d.
7. Lee, Y.; Kwon, J.; Hwang, E.; Ra, C.-H.; Yoo, W.J.; Ahn, J.-H.; Park, J.H.; Cho, J.H. High-Performance Perovskite-Graphene Hybrid Photodetector. *Adv. Mater.* **2014**, *27*, 41–46, https://doi.org/10.1002/adma.201402271.
8. Sun, Z.; Aigouy, L.; Chen, Z. Plasmonic-enhanced perovskite–graphene hybrid photodetectors. *Nanoscale* **2016**, *8*, 7377–7383, https://doi.org/10.1039/c5nr08677a.
9. Chen, Z.; Kang, Z.; Rao, C.; Cheng, Y.; Liu, N.; Zhang, Z.; Li, L.; Gao, Y. Improving Performance of Hybrid Graphene–Perovskite Photodetector by a Scratch Channel. *Adv. Electron. Mater.* **2019**, *5*, https://doi.org/10.1002/aelm.201900168.
10. Wang, Y.; Zhang, Y.; Lu, Y.; Xu, W.; Mu, H.; Chen, C.; Qiao, H.; Song, J.; Li, S.; Sun, B.; et al. Hybrid Graphene-Perovskite Phototransistors with Ultrahigh Responsivity and Gain. *Adv. Opt. Mater.* **2015**, *3*, 1389–1396, https://doi.org/10.1002/adom.201500150.
11. Spina, M.; Lehmann, M.; Náfrádi, B.; Bernard, L.; Bonvin, E.; Gaál, R.; Magrez, A.; Forró, L.; Horváth, E. Microengineered $CH_3NH_3PbI_3$Nanowire/Graphene Phototransistor for Low-Intensity Light Detection at Room Temperature. *Small* **2015**, *11*, 4824–4828, https://doi.org/10.1002/smll.201501257.
12. Kang, D.-H.; Pae, S.R.; Shim, J.; Yoo, G.; Jeon, J.; Leem, J.W.; Yu, J.S.; Lee, S.; Shin, B.; Park, J.-H. An Ultrahigh-Performance Photodetector based on a Perovskite-Transition-Metal-Dichalcogenide Hybrid Structure. *Adv. Mater.* **2016**, *28*, 7799–7806, https://doi.org/10.1002/adma.201600992.
13. Ma, C.; Shi, Y.; Hu, W.; Chiu, M.; Liu, Z.; Bera, A.; Li, F.; Wang, H.; Li, L.; Wu, T. Heterostructured WS_2/$CH_3NH_3PbI_3$ Photoconductors with Suppressed Dark Current and Enhanced Photodetectivity. *Adv. Mater.* **2016**, *28*, 3683–3689, https://doi.org/10.1002/adma.201600069.
14. Khan, A.; Yu, Z.; Khan, U.; Dong, L. Solution Processed Trilayer Structure for High-Performance Perovskite Photodetector. *Nanoscale Res. Lett.* **2018**, *13*, 399, https://doi.org/10.1186/s11671-018-2808-7.
15. Subramanian, A.; Akram, J.; Hussain, S.; Chen, J.; Qasim, K.; Zhang, W.; Lei, W. High-Performance Photodetector Based on a Graphene Quantum Dot/$CH_3NH_3PbI_3$ Perovskite Hybrid. *ACS Appl. Electron. Mater.* **2019**, *2*, 230–237, https://doi.org/10.1021/acsaelm.9b00705.
